# Raynaud's Disease

**Published:** 1900-09

**Authors:** 


**Raynaud's Disease.** By THOMAS KIRKPATRICK MONRO, M.D. Pp. xii., 251. Glasgow: James Maclehose & Sons. 1899.

This essay contains a complete account of our present knowledge of Raynaud's disease. The history of its discovery, etiology, morbid anatomy, and treatment, are fully discussed in the light of practically all the cases which have hitherto been published.

One or two points, perhaps, seem open to criticism. It is a little to be regretted that the terms originally used by Raynaud, "local syncope" and "local asphyxia," are still employed when we now have more descriptive ones at command. In the discussion of paroxysmal hæmoglobinuria, it is not pointed out that hæmatogenous jaundice is in reality a urobilinæmia. And Haig's now discredited uric acid theories are stated without a word of criticism. But such matters are really trifling in comparison with the wealth of accurate statements and generalisations in which the book abounds, and which make it a most useful and valuable guide to a little-understood group of diseases.

